# The Bristol Royal Infirmary Impasse

**Published:** 1907-10-05

**Authors:** 


### HOSPITAL ADMINISTRATION.

### CONSTRUCTION AND ECONOMICS.

### THE BRISTOL ROYAL INFIRMARY IMPASSE.

GRAVE DISSATISFACTION OF HONORARY MEDICAL STAFF.

At the half-yearly meeting of the Governors of the Bristol Royal Infirmary, held on September 24 at the hospital, the following resolutions were carried, after an animated discussion, by 47 votes to 26:—

Resolved: "That all appointments to the honorary staff to be made subsequent to the passing of this rule shall be held subject to the following regulations, in lieu of those contained in Rule 35."

"Rule 36: No member of the honorary staff shall hold any union or club appointment. No member of the full staff shall hold any other professional public appointment other than professorship or lectureship at any university, college, or school. No member of the assistant staff shall hold any other general hospital appointment, nor more than one special hospital appointment.

"That the full physicians shall limit their practice to medical work. That the full surgeons shall limit their practice to surgical work. That each of the specialists shall limit his practice to his speciality."

Several of the past and present members of the medical staff spoke at the meeting, among them three of the surgeons, Mr. Munro Smith, Mr. James Swain, and Mr. Paul Bush, C.M.G.; the consulting physician, Dr. Shingleton Smith, the obstetric physician, Dr. Walter Swayne, Dr. Harrison, consulting physician to the Bristol General Hospital, and Mr. T. Carwardine, one of the out-patient surgeons. All these gentlemen opposed the resolutions, on the ground that they objected to any legislation on the disposition of the time of individual members of the honorary medical staff outside the infirmary walls. The staff contend that if they do their work well, and no fault is found with it so far as the infirmary was concerned, they had a perfect right to dispose of their own time apart from the infirmary in any way they thought fit. Mr. Munro Smith declared that a very grave mistake had been made by a member of the staff, Dr. Arthur B. Prowse, who had sent some resolutions to the President of the Infirmary which he thought would meet the case. Dr. Prowse suggested that the end in view might be attained without any friction by rules applicable to the whole honorary staff as follows:—(1) That no member of the honorary staff shall hold any union or club appointment. (2) That no member of the full staff shall hold any other professional public appointment. (3) That no member of the assistant staff shall hold more than one hospital appointment. (4) That the full physicians shall limit their practice to medical work. (5) That the full surgeons shall limit their practice to surgical work. (6) That each of the specialists shall limit his practice to his speciality.

As explained by the President, the Committee of Management were exceedingly anxious to do everything possible, consistent with their duty to the Governors, to fall in with any views of the medical staff. They therefore unanimously adopted the suggestions of Dr. Prowse, with a modification to Clause 3, which the Committee drafted as follows:—"That no member of the assistant staff shall hold any other general hospital appointment or more than one special hospital appointment." Thereupon the staff, as explained by the President, resolved that regulation 2 restraining members of the full staff from holding any other professional appointment was "infeasible," and that certain exceptions were desirable. The staff further expressed the hope that regulation 4 would not refer to any present member of the honorary staff, and noted that the rule was not to be retrospective.

The matter was then brought before the Committee once more, when the honorary staff were represented by Dr. Watson Williams. The President stated that Dr. Williams, as representative of the faculty, acquiesced in the proposed new rules, if he could not get anything more. In the result the Committee adopted the modification permitting the staff to hold professorships or lectureships at any university, college, or school. Dr. Williams then, according to the President, wanted to review the whole position, and to induce them to make a number of variations, but the Committee considered they had done all they could by rescinding their original resolution and accepting the staff's proposals with the variations just indicated. The President further read a letter from Dr. Ogilvy to show that the faculty were well satisfied at the time with the resolutions as they stand. In the course of his letter Dr. Ogilvy stated "I am very pleased that we have been able to settle our little differences so amicably, and would have been most happy to have voted for your new resolution if I had been present, as it represents the wish of the majority, and the large majority, I am glad to say, of the faculty." The President further contended, in the course of his speech, that the Bristol General Hospital had had a rule for many years requiring the sanction of the Committee to any additional medical appointment taken by a member of the honorary staff, and that "the great hospitals and infirmaries of Birmingham, Liverpool, Leeds, and Manchester all had limitations, more or less following upon the lines embodied in the resolution." It would further appear, if Dr. Ogilvy is right, that the decision of the Governors represents the views of the majority of the faculty.

We have evidence, however, that certain members of the staff resent very strongly the idea that a consulting physician or surgeon should limit his practice outside the infirmary in accordance with the wishes of a number of laymen, whose sanction he must first obtain as to how he is to employ his spare hours. They maintain that no lay committee ought to claim to sit as arbitrators of the social and professional life of members of the staff outside and beyond the duties of their office in the institution to which they are attached. Special objection is taken by the dissentients to the restrictions placed upon the assistants, as calculated to restrict the energies of enthusiastic young physicians and surgeons, which would compel them to let pass opportunities open to them, and so to waste a considerable portion of their early years.

The objection of most substance seems to lie in the contention that the resolutions as adopted by the Governors would prevent members of the honorary staff of the Bristol Royal Infirmary from holding the Presidency of the Royal College of Physicians, of the Royal College of Surgeons, the post of physician or surgeon to Clifton College, both confined to pure physicians and surgeons, appointments under the Midwifery Act, the office of visitor appointed by Commissioners to Asylums, and even such an honourable appointment as that of physician or surgeon to His Majesty the King. All military instincts in the staff must also necessarily be suppressed, as none of its members, under the resolutions as they stand, can ever be a surgeon to a volunteer corps. These contentions are canvassed, we believe, by the President, Sir George White, but they should in any case be considered very carefully, and provided for before the resolutions adopted on September 26 are brought up for confirmation at the special meeting of Governors to be held shortly. Meanwhile, we shall be glad to hear that unanimity and agreement have been arrived at between the faculty and the governing body of the Royal Infirmary at Bristol. The excellent spirit displayed by the speakers during the discussion at the meeting of Governors leads us to hope that such a settlement will in fact be come to very shortly.

